# Change and consistency in *Acta Radiologica* over 100 years

**DOI:** 10.1177/02841851211054174

**Published:** 2021-10-22

**Authors:** Mats Geijer, Henrik S Thomsen

**Affiliations:** 1 Department of Radiology, Institute of Clinical Sciences, 70712 Sahlgrenska Academy, University of Gothenburg, Gothenburg, Sweden; 2 Department of Radiology, Region Västra Götaland, Sahlgrenska University Hospital, Gothenburg, Sweden; 3 Department of Clinical Sciences, Lund University, Lund, Sweden; 4 University of Copenhagen, Copenhagen University Hospital, Herlev & Gentofte, Herlev, Denmark

**Keywords:** History, radiology, publishing

## Abstract

*Acta Radiologica* celebrates its 100th anniversary in 2021. In this article, the foundation of the journal and its editors are described. During 100 years, the manuscript structure changed from single-author verbose monographs to multi-author collaborations on statistically analyzed research subjects. The authorship changed from purely Nordic authors to a truly international cadre of authors, and the size of the journal increased considerably, in issues per year, printed pages, and published articles per year. The Foundation of Acta Radiologica has been able to give out two prizes, the Xenia Forsselliana and the Acta Radiologica International Scientific Prize for the best manuscripts each year. The increasing submissions of manuscripts is an indication that Acta Radiologica will continue to publish important scientific results for many years to come.

## Introduction

The radiology journal *Acta Radiologica* has now been in print for 100 years. In the current issue, the evolution of radiology during these years is exemplified in several articles, from both clinical and technical points of view. The clinical development is described in three articles. Bone radiography, the first application for X-rays which has since morphed into musculoskeletal radiology, is described by Geijer et al. (1). The history of neuroradiology, slightly younger than musculoskeletal radiology, is described from the Stockholm perspective by Hindmarsh and Kaijser (2). Breast radiology, with the first clinical images being taken in the 1950s, is described by Zackrisson and Andersson (3). The development of imaging modalities is exemplified by the introduction and evolution of magnetic resonance (MR) techniques and ultrasonography. While Odeblad and Lindström had already reported on MR spectroscopy in 1955 (4), the first commercially available system for imaging of the human body was introduced in 1980, described by Smith in the current issue of *Acta Radiologica* (5). Ultrasonography, with imaging of the human body beginning in the 1950s, is described by Nielsen et al. (6). Contrast media are now an integral and irreplaceable part of all kinds of radiology. Its discoveries, evolution, and applications today are described by Nielsen and Thomsen (7). Last, but not least, we find the backbone of today's radiology, information technology solutions, as described by Reponen and Niinimäki (8).

Below, a brief description is given of the general changes in the journal. The historically interested reader is also directed to previous historical reports on Swedish radiology in *Acta Radiologica* by Erik Boijsen given at the 75th jubilee (9), in the Supplementum 434 in 2008, edited by Anders Hemmingsson at the celebration of the 85th jubilee (10), and by Arnulf Skjennald in the current issue summing up the next 15 years (11).

## The founding of *Acta Radiologica*

When the Great War ended in 1918, the world (mostly Europe) had experienced one of the deadliest conflicts in history over five years, the toll on human lives being exacerbated by the pandemic Spanish flu from 1918 to 1920. Even though the Nordic countries had managed to keep out of the conflict (except Finland, which was part of the Russian Empire until December 1917), the Great War and the pandemic must have had a great impact on life in Scandinavia as well. It seems reasonable that 1921 should be the birth-year for *Acta Radiologica* after some time for planning and discussion after the end of the war. Before the founding of *Acta Radiologica*, Nordic radiologists had their manuscripts published in different journals. For example, Gösta Forssell's first publication in radiology, on the movements in the human wrist, was printed in *Skandinavisches Archiv für Physiologie* in 1902 (12), which today is published as *Acta Physiologica*.

Gösta Forssell was the initiator of the Swedish Society of Medical Radiology as well as of the Nordic Society of Medical Radiology. He convinced his Nordic colleagues that a Nordic radiology journal should be founded, where Nordic radiologists could publish their works. It should also include works by non-Nordic authors to inform Nordic readers of events outside the Nordic countries. After intense discussions with colleagues from the Nordic countries, it was decided that Forssell should be the chief editor with an editorial office in Stockholm, Sweden, and that each Nordic country should have a local editorial office where the local editor could receive manuscripts from his own country (9). In a few years, *Acta Radiologica* had expanded such that the Dutch Radiological Society in 1923 and the Swiss Society in 1926 joined the editorial board on the same conditions as the societies from the Nordic countries. This broader European collaboration ended shortly after World War II in 1956, when radiology began to expand rapidly into different directions of subspecialization and technological development.

From the beginning, Gösta Forssell personally financed and published *Acta Radiologica*, and thus was the more or less formal owner of the journal. This changed in 1939, when Gösta Forssell wrote that “…the owner of *Acta Radiologica* has never been formally settled. The undersigned that has been authorized as the publisher of the journal and during all years responsible for its economy should under the present circumstances be the owner of the journal and its economic belongings … I suggest that the Nordic Countries of radiology form a society that owns and distribute the journal …” (10). A Society of Acta Radiologica was thus formed, and the finances for *Acta Radiologica* were transferred to the society.

## Editors

**Fig. 1.**
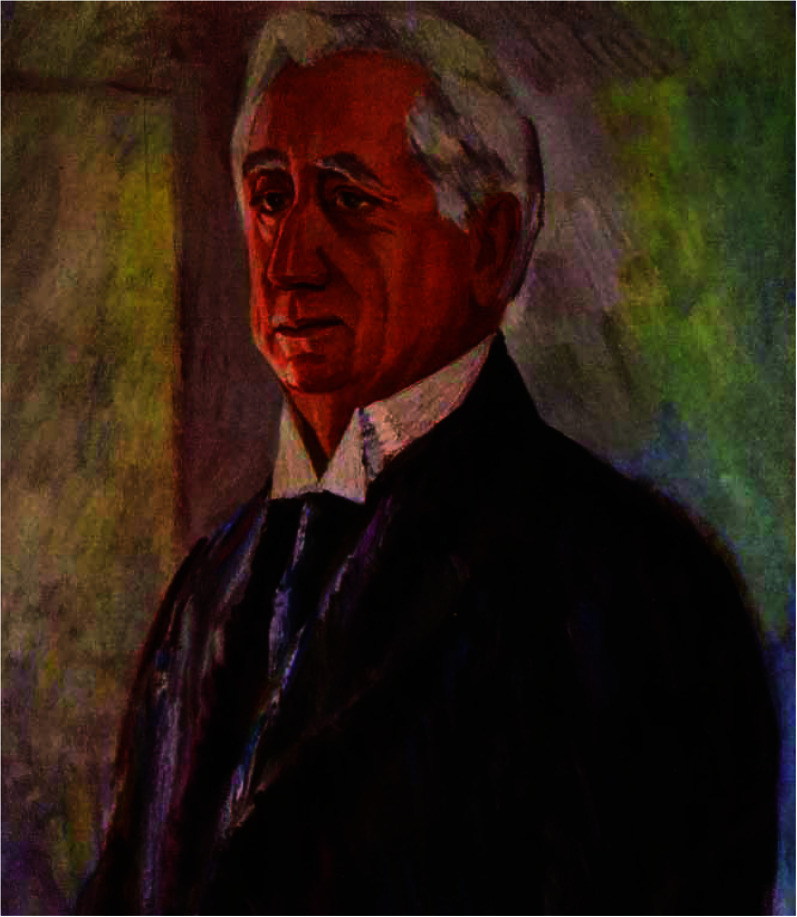
Gösta Forssell in 1941, portrait by Isaac Grünewald. From (62).

In 1921, the first issue of *Acta Radiologica* was published (13), making *Acta Radiologica* one of the oldest published radiological journals still in print, as well as one of the oldest medical journals, still published under its original name. Since 1921, *Acta Radiologica* has had seven editors (Table 1). Acta *Radiologica* was conceived, founded, and, for nearly three decades, edited by Gösta Forssell until his death in 1950 (14). Gösta Forssell (Fig. 1) was the world's first professor of radiology in Stockholm, Sweden. Much of his time was devoted to organizing the Radiumhemmet hospital and King Gustaf Vth Jubilee Clinic for oncologic patients. In 1956, Åke Åkerlund, in a Supplementum to *Acta Radiologica*, published an extensive review of Forssell's life and work (15) including a bibliography of Forssell's more than 200 published works. Most of Forssell's academic work was published before *Acta Radiologica* was founded, and his most important work published in *Acta Radiologica* deals with teaching in radiology where he edited and published a supplement with contributions from around the world in 1930 (16). Åke Åkerlund's words of remembrance after Forssell's death at the meeting of the Swedish Medical Association in November 1950 (17) amply illustrate the primitive conditions and early pioneer work of Gösta Forssell; here is a description of Forsell's first work on radiology from 1902 (12): “…(it) was inspired by Forssell's intimate co-operation with Prof. Erik Müller at the Institute of Anatomy. It was worked out during Forssell's first roentgen post as assistant at Dr T. Stenbeck's private Roentgen Institute in Stockholm … Forssell worked under primitive conditions without direct daylight on a kind of built-up intermediate floor at the back of the little room. By careful detailed analysis of a great number of roentgenograms taken in different positions of the wrist-joint and by comparing them carefully with anatomical specimens, Forssell demonstrated for the first time in this work on living material, the complicated mechanism of movement and the relative changes in position of the small bones of the wrist-joint.”

**Table 1.** The editors of *Acta Radiologica*.

1921–1950	Gösta Forssell (1876–1950), Sweden
1950–1951	Elis Berven (1885–1966), Sweden
1951–1982	Erik Lindgren (1905–2005), Sweden
1983–1992	Erik Boijsen (1922–2017), Sweden
1993–2002	Anders Hemmingsson (1935–2017), Sweden
2003–2017	Arnulf Skjennald (1944–), Norway
2018–present	Henrik S Thomsen (1953–), Denmark

**Fig. 2.**
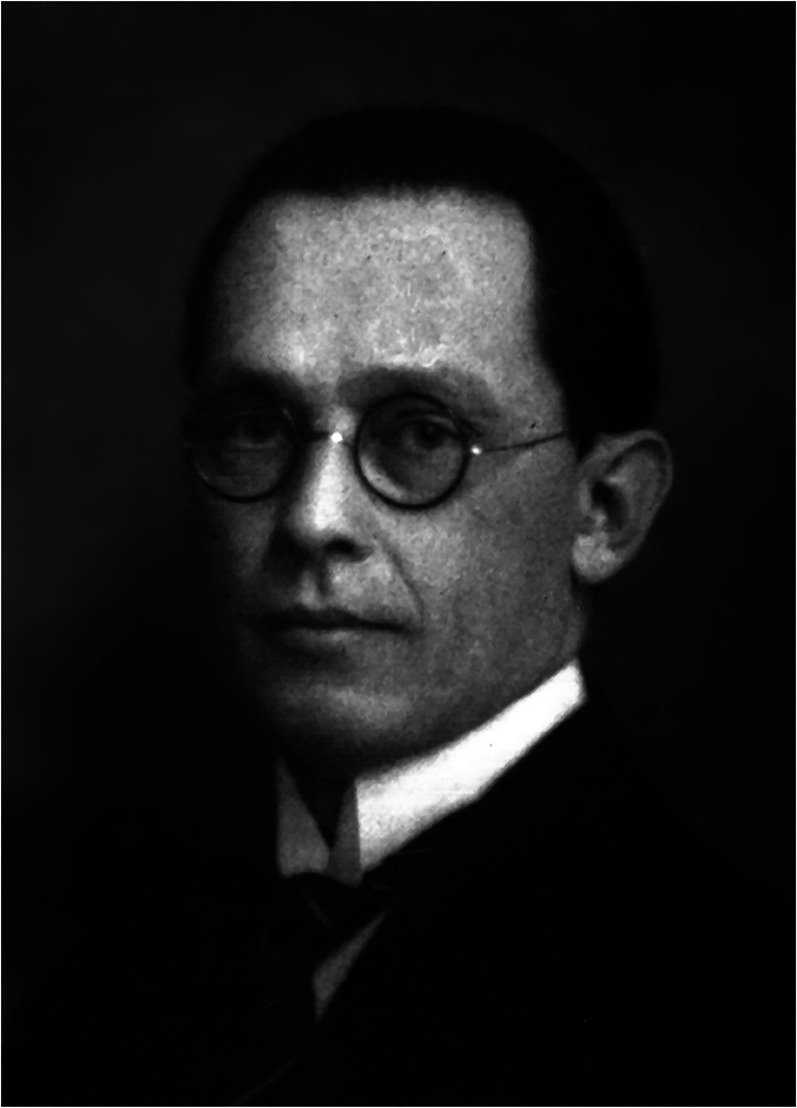
Elis Berven. From Wikipedia.

After Forssell's death, Elis Berven in Stockholm (Fig. 2), professor of radiotherapy (18), acted as temporary editor from 1950 to 1951, when Erik Lindgren could be elected by the board (19). Elis Berven was foremost a therapeutic radiologist treating malignant tumors, mainly in the head and neck (20–22) and was a world-renowned authority in this field.

**Fig. 3.**
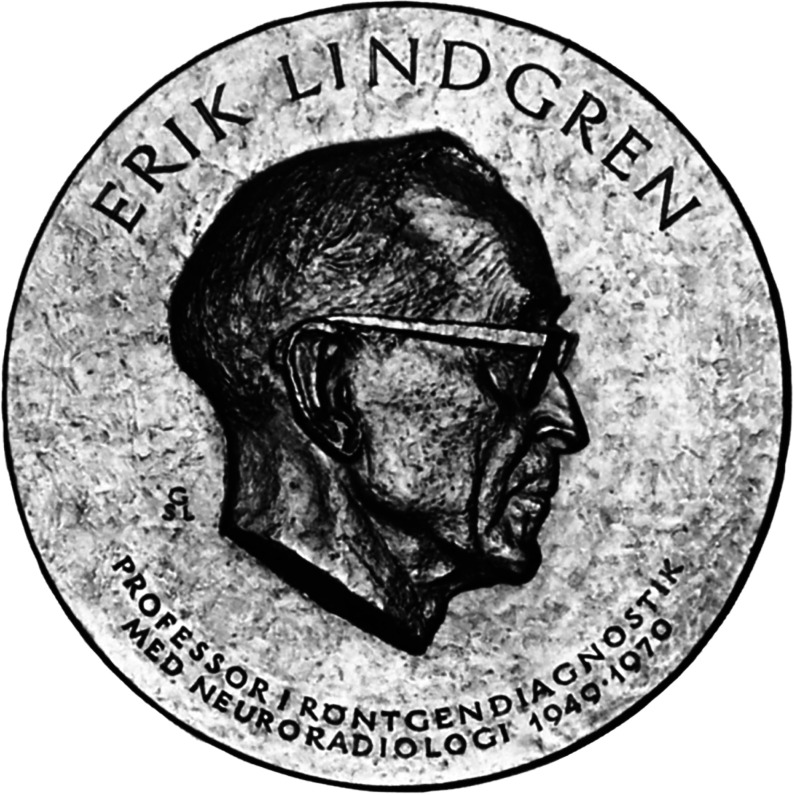
Erik Lindgren. From (19).

Erik Lindgren (Fig. 3) became editor in 1951 and would go on to edit *Acta Radiologica* for an astounding 32 years. During Lindgren's time, radiology evolved dramatically in angiographic techniques, and almost all parts of the body became accessible for the catheter. In *Acta Radiologica*, the new catheter replacement technique by Seldinger was presented in 1953 (23), probably the most cited article ever published in *Acta Radiologica*. Lindgren's importance for Swedish radiology and for *Acta Radiologica* cannot be overstated, as is demonstrated in the article about Swedish neuroradiology in the current issue of *Acta Radiologica* (2). Apart from the development of neuroradiology, other fields of angiography were of interest, such as angiocardiography (24,25), gynecologic and obstetric radiology (26,27), and renal angiography (28,29). Not least importantly, publications on technical development included catheter development (30–32), the film changer (33,34), and the pressure injector (35).

**Fig. 4.**
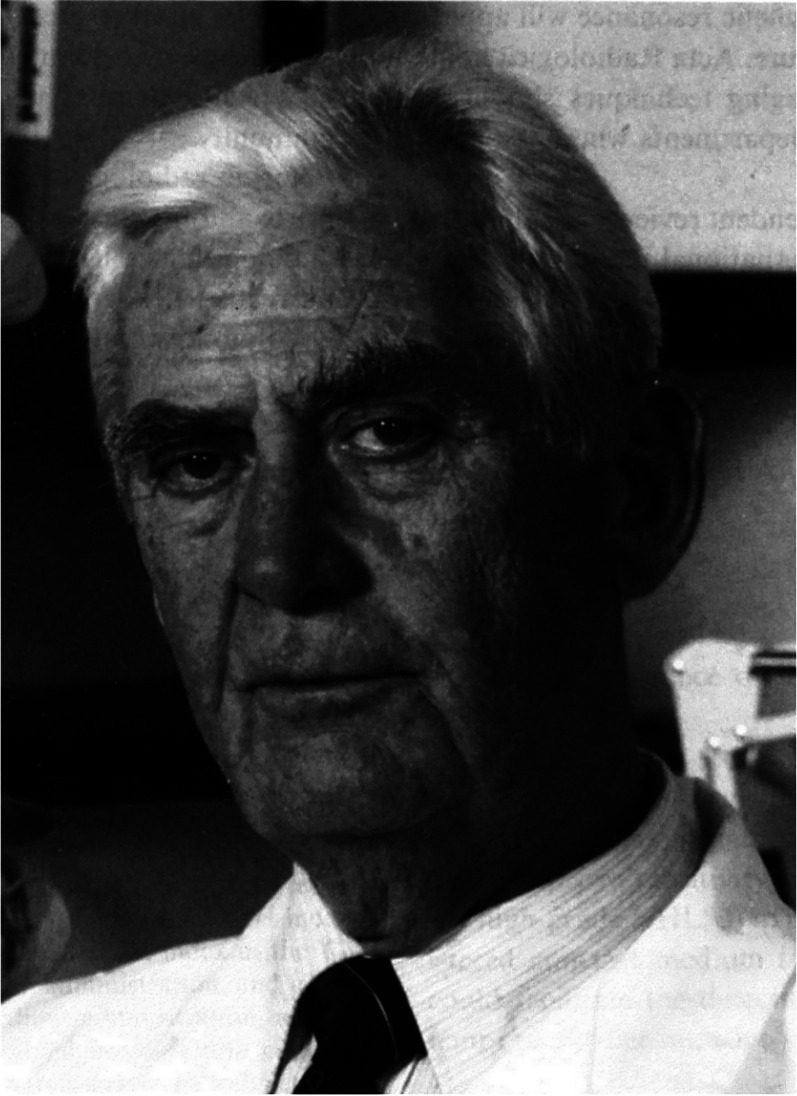
Erik Boijsen. From (59).

**Fig. 5.**
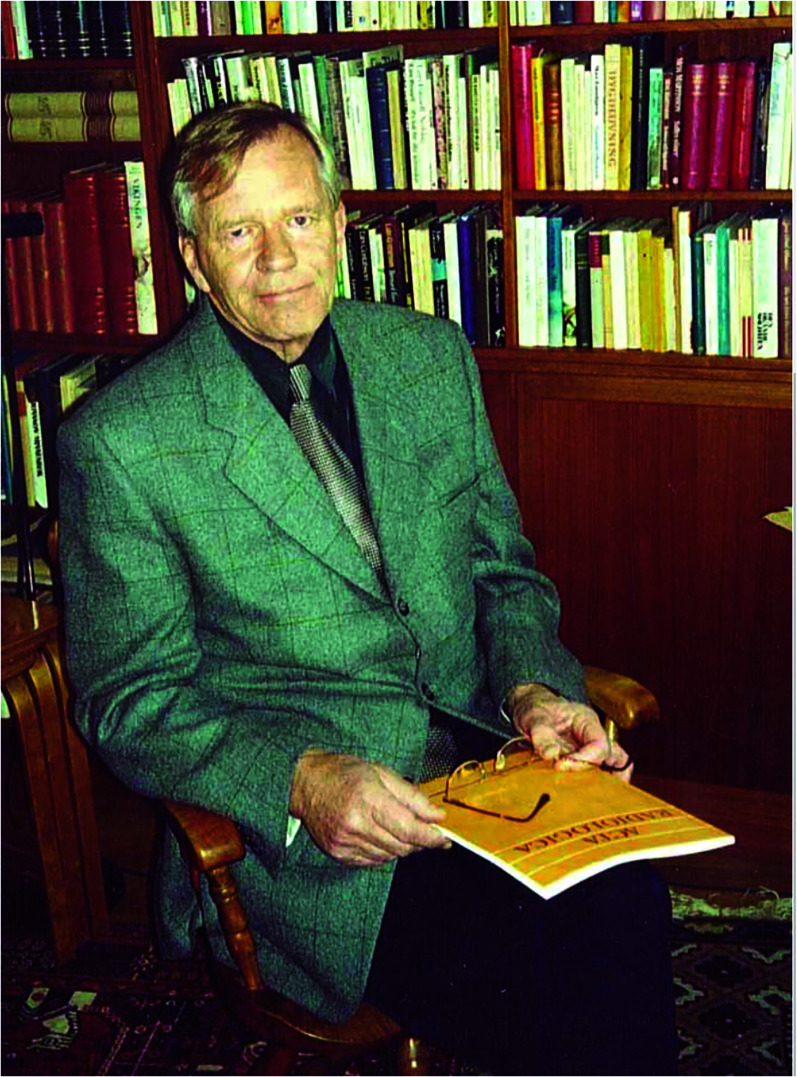
Anders Hemmingsson. From (10).

**Fig. 6.**
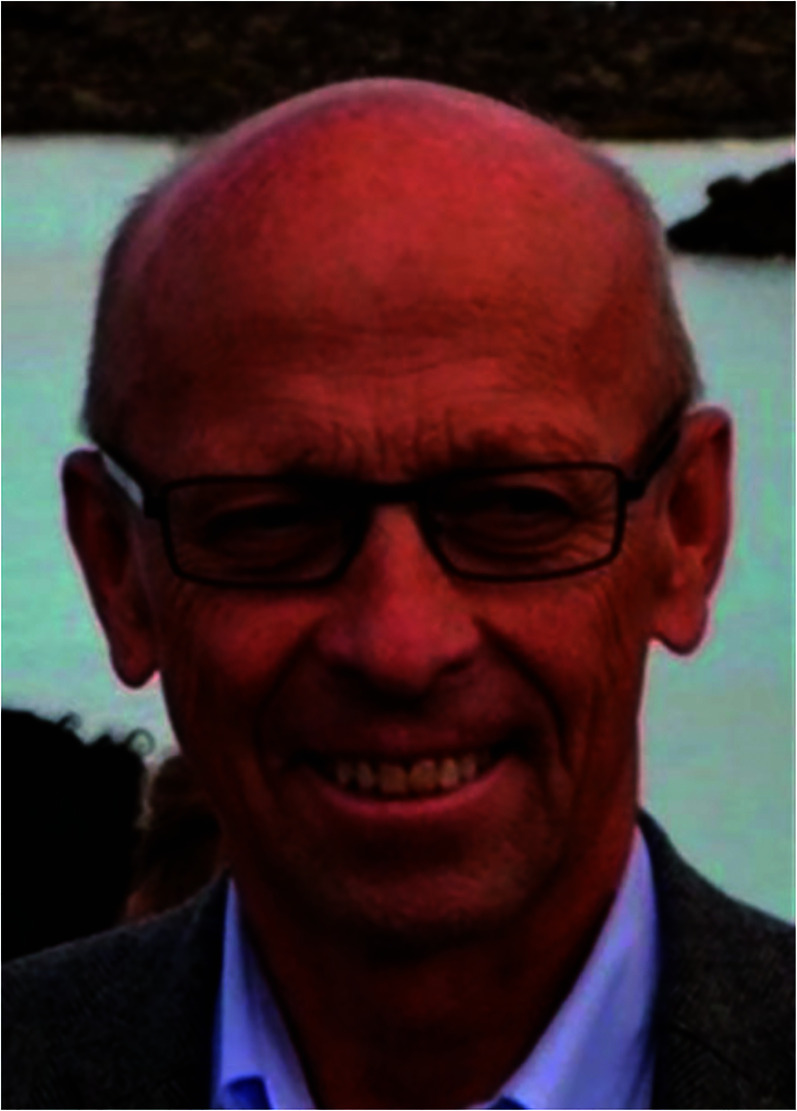
Arnulf Skjennald. From (37).

**Fig. 7.**
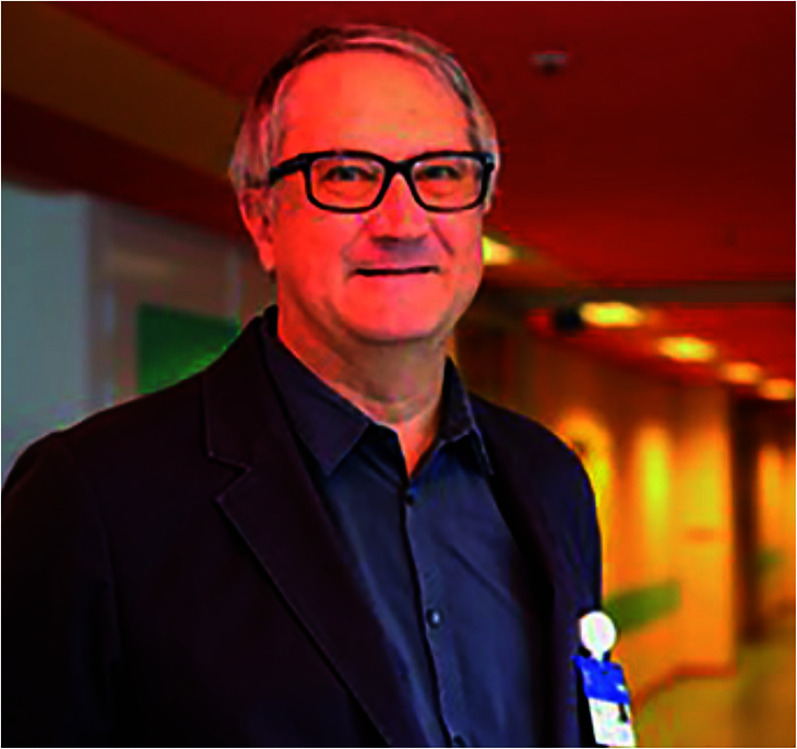
Henrik S Thomsen. From (38).

After Erik Lindgren, Erik Boijsen (Fig. 4) became editor for the next decade in 1983 (36), followed by Anders Hemmingsson (Fig. 5) for another decade in 1993 (36). Arnulf Skjennald (Fig. 6) became editor in 2003, a task he fulfilled for 15 years until he retired as chief editor in 2017 (37) and the current chief editor, Henrik S Thomsen (Fig. 7) from Copenhagen, Denmark, took up the post in 2018 (38). During Erik Boijsen's time, we can witness the explosion of computed tomography (CT) of the brain and body, its associated techniques, articles about radiation protection, the emergence of ultrasonography as an important diagnostic and therapeutic modality, and about the use of contrast media. The rapid development of CT is shown in the exploration of its usefulness in all organ systems, such as early examples of investigation of pulmonary embolism (39), high resolution CT of cystic fibrosis (40), hepatic tumor imaging (41), the use of CT for biopsy guidance (42), and many more. Anders Hemmingsson was intimately associated with MR research in Uppsala in Sweden, and the emergence of MR imaging (MRI) techniques to become a dominant force among the imaging modalities is evident from the increasing number of MR publications in *Acta Radiologica* during his editorship (5). Examples from his period as editor include the advantages of lumbar spine imaging with MRI (43), and the usefulness of MRI for tumor evaluation (44), occult trauma evaluation (45), ligament and tendon evaluation (46,47) and the reporting of new techniques such as MR angiography (48) and diffusion-weighted imaging (49), among others. During Arnulf Skjennald's time as editor, CT and MRI became mature techniques, and the emergence of artificial intelligence as something that will come to play an important role in radiology in the future is demonstrated in examples from breast (50–52), abdominal (53,54), and chest (55) investigations.

## Manuscripts

A century ago, radiologists were as much clinicians and oncologists as they were diagnostic radiologists, which is reflected in the pages of *Acta Radiologica*. About half of the articles from the first three decades are dedicated to treatment. In addition, the technical developments and inventions of, for example, Lysholm's skull table (56), catheters (30), and film changers (34) were published, as were papers purely dedicated to radiation physics, not least by the legendary Rolf Sievert of the eponymous radiation measurement unit (57) who wrote extensively on radiation and radiation protection from the first issue in 1921 (58) well into the 1950s. Eventually, with radiologic treatment and diagnosis taking different paths and oncology moving away from the radiology department, in 1963 the journal was split into one journal dedicated to diagnostic radiology and the other to therapeutic radiology, i.e. mainly oncology. In 1987, the foundation that owned the journals separated into two (59). Consequently, reporting of diagnostic radiology continued from *Acta Radiologica* into *Acta Radiologica: Diagnosis* in 1963, and after a further name change into *Acta Radiologica (Stockholm, Sweden: 1987)* in 1987, which still is the official name. The reporting of therapeutic radiology moved from *Acta Radiologica* into *Acta Radiologica: Therapy, Physics, Biology* in 1963, and after further name changes with various suffixes to *Acta Radiologica* in 1978, 1980, and 1984, eventually acquired the name *Acta Oncologica (Stockholm, Sweden)* in 1987.

Due to the separation of *Acta Radiologica* into two publications dedicated to different subjects in 1963, volume numbering had to restart, with volume 58 in 1962 being the last volume of *Acta Radiologica*, and volume 1 in 1963 being the first in the new series of *Acta Radiologica: Diagnosis* and *Acta Radiologica: Therapy, Physics, Biology*. Sometimes the original *Acta Radiologica* is referred to as the “old series.”

The evolution of manuscript structure is also evident when perusing *Acta Radiologica*. The early articles are purely one-author products, probably often the printed version of a speech given at a medical convention. The first multi-author (i.e. three or more authors) paper appeared in 1925 (60), whereas today's papers are nearly always multi-author collaborations with the number of authors not seldom exceeding ten. This reflects the increasing complexity in medical research and increased needs for meriting, resulting in the change from narrative articles without illustrations to structured articles with impressive statistical calculations. The length of the papers is also different. Today, most papers are almost invariably 7–10 pages long due to printing costs with a limitation on the allowed number of words, tables, and figures. The possibility to reproduce diagnostic images from all modalities, illustrate the paper with statistical graphs, and reproduce images in color has reduced the need for long verbose descriptions that were previously needed. Nevertheless, for the first five volumes (1921–1926), the mean number of pages per article is 12 (median 9), but the range is much wider than today, from 1–2 pages to a maximum of 63 pages. All meeting reports, editorials, and obituaries were omitted from these calculations. One striking fact is the lack of illustrations early on, where high-quality photographic reproduction of radiographic were printed on separate sheets that were interfoliated at appropriate locations in the printed issue and the articles themselves at most included drawings of the radiographs that were technically easier to reproduce. The high-quality reproductions have not always been scanned for the articles available online.

## Printed journal

From being a radiology journal dedicated to serving a Nordic audience and Nordic authors, *Acta Radiologica* has evolved into a truly international journal. Several factors can be attributed to this development. The leading members of the Nordic radiological societies were personally deeply involved in the founding of *Acta Radiologica*. With today's much higher number of radiologists and more distributed areas of responsibility, a few leading radiologists are no longer in the same way personally responsible for publication and content as in the beginning; the gradually increasing number of radiologists, researchers, and manuscripts over the years has increased the inflow of manuscripts from all parts of the world, and not least, the evolution of communication across the world has made the submission and handling of manuscripts easier. During the last 100 years, there has been a revolution in correspondence and manuscript handling, from regular mail delivered by car, bus, train, and boat to intercontinental flights, telefax, email, and today online submission and manuscript handling. This internationalization of communication is clearly reflected in the increasing breadth of nationalities of authors, from nearly solely Nordic authors in the early volumes evolving to encompass first European authors, then also North American authors, and Asian authors from mainly Japan, the Republic of Korea, and lately the People’s Republic of China.

English was not as dominant a scientific language 100 years ago as it is today, which is reflected in the early volumes. From the beginning, it was decided that the manuscripts published in *Acta Radiologica* should be written in English, German, or French, with summaries in the other languages. Many early authors wrote articles in all three languages at different times. Gradually, the English language acquired a clear dominance, and the last non-English paper was published in 1972 (61). Summaries in the other languages ended in 1980.

Over the years, the journal has increased considerably in size, both in number of issues and pages per year. The 100th volume in 2020 comprised 12 issues consisting of 1726 printed pages and 263 articles. There were 1123 original submissions with an acceptance rate of 26%. The submissions originated from 51 countries, the top five being China, Turkey, Republic of Korea, Japan, and Germany. The impact factor in 2019 was 1.635, which is reasonable for a radiology journal. The five most cited articles from 2018 and 2019 in 2020 are shown in Table 2. With publishing having switched over almost entirely to digital publication, downloads of full-text articles are increasing yearly. The five most downloaded articles in 2020 are shown in Table 3.

**Table 2.** The five most downloaded articles during 2020.

No. of citations	Authors	Title	Reference
3300	Yoon HM, Byeon S-J, Hwang J-Y, Kim JR, Jung AY, Lee JS, Yoon H-K, Cho YA	Sacrococcygeal teratomas in newborns: a comprehensive review for the radiologists	Acta Radiol 2018;59(2):236–246
2602	Yaniv G, Katorza E, Tsehmaister Abitbol V, Eisenkraft A, Bercovitz R, Bader S, Hoffmann C	Discrepancy in fetal head biometry between ultrasound and MRI in suspected microcephalic fetuses	Acta Radiol 2017;58(12):1519–1527
1364	Park J-H, Kim KY, Song H-Y, Cho YC, Kim PH, Tsauo J, Kim MT, Jun EJ, Jung H-Y, Kim S-B, Kim JH	Radiation-induced esophageal strictures treated with fluoroscopic balloon dilation: clinical outcomes and factors influencing recurrence in 62 patients	Acta Radiol 2018;59(3):313–321
1195	Park JW, Ko KH, Kim E-K, Kuzmiak CM, Jung HK	Non-mass breast lesions on ultrasound: final outcomes and predictors of malignancy	Acta Radiol 2017;58(9):1054–1060
1149	van Zelst JC, Tan T, Mann RM, Karssemeijer N	Validation of radiologists’ findings by computer-aided detection (CAD) software in breast cancer detection with automated 3D breast ultrasound: a concept study in implementation of artificial intelligence software	Acta Radiol 2020;61(3):312–320

**Table 3.** The top five cited articles in 2020 from the publication years 2018–2019.

Title	Authors	Year
Pancreatic neuroendocrine tumor: prediction of the tumor grade using CT findings and computerized texture analysis	Choi TW, Kim JH, Yu MH, Park SJ, Han JK	Acta Radiol 2018;59(4):383–392
Accuracy of high b-value diffusion-weighted MRI for prostate cancer detection: a meta-analysis	Godley KC, Syer TJ, Toms AP, Smith TO, Johnson G, Cameron D, Malcolm PN	Acta Radiol 2018;59(1):105–113
Sacrococcygeal teratomas in newborns: a comprehensive review for the radiologists	Yoon HM, Byeon S-J, Hwang J-Y, Kim JR, Jung AY, Lee JS, Yoon H-K, Cho YA	Acta Radiol 2018;59(2):236–246
Quantitative and qualitative MRI evaluation of cerebral small vessel disease in an elderly population: a longitudinal study	Nylander R, Fahlström M, Rostrup E, Kullberg J, Damangir S, Ahlström H, Lind L, Larsson E-M	Acta Radiol 2018;59(5):612–618
Accuracy of the diagnostic evaluation of hepatocellular carcinoma with LI-RADS	Liu W, Qin J, Guo R, Xie S, Jiang H, Wang X, Kang Z, Wang J, Shan H	Acta Radiol 2018;59(2):140–146

In the Supplements to *Acta Radiologica*, many important discoveries and inventions have been published. For many years, most Swedish dissertations were published. A thorough description of the Supplements has been given by Anders Hemmingsson in the supplement published at the 85th anniversary (10).

## Important publications

Apart from all the papers referenced in the articles in the current issue of *Acta Radiologica*, Erik Boijsen listed important publications from Swedish radiology at the 75th anniversary (9) and Anders Hemmingsson from the other Nordic countries as well (10).

## Scientific prizes

When Gösta Forssell retired as professor of radiology and celebrated his 65th birthday in 1941, he was honored by having a portrait of him uncovered (Fig. 1) (62), painted by the famous Swedish painter Isaac Grünewald. He was also honored by a publication from 120 radiologists in 13 countries named Xenia Forsselliana (63). It was published as issues 1–2 and 5–6 of *Acta Radiologica* in 1941 (volume 22). Further, a fund was set up in his name, also named Xenia Forsselliana. In 1993, it was decided that a stipend of the fund each year should be given to the best article published in *Acta Radiologica* by a Nordic author as the Xenia Forsselliana Prize (62).

Realizing that *Acta Radiologica* had grown to have a broad international field of authors, the Board of the Foundation in 2015 decided to establish a prize also for the best non-Nordic contribution to *Acta Radiologica* each year, the Acta Radiologica International Scientific Prize. Recipients of both prizes receive a diploma and the same prize sum. All recipients are also invited to the next Nordic radiology conference to present the award-winning paper (11).

The lists of award-winners for both prizes, up until 2020, can be seen in the article by Arnulf Skjennald in the current issue of *Acta Radiologica* (11).

## Offspring

Case reports were discontinued from the printed pages of *Acta Radiologica* in 2010 since there was a high rejection rate—sometimes as high as 80%—and since case reports, being rarely cited, were detrimental to the citation index. There was, however, a large interest among the readers in case reports, and it was decided to establish a daughter journal dedicated specifically to case reports; *Acta Radiologica Short Reports*, further described by Arnulf Skjennald in the current issue (11). The first issue was published in February 2012. In time, it turned out that not only case reports, but also high-quality scientific studies, were published there, and a decision was taken in 2015 to change the name to *Acta Radiologica Open*.

## Future

Now 100 years old, *Acta Radiologica* looks to a bright future. Despite strong competition from other general radiology journals, it is keeping its place among the top, with a strong citation index and a vigorous inflow of new manuscripts. After 100 years in print, the current issue may be one of the last, before *Acta Radiologica* switches over entirely to electronic publishing.
